# Stromal area differences with epithelial-mesenchymal transition gene changes in conjunctival and orbital mucosa-associated lymphoid tissue lymphoma

**DOI:** 10.3389/fonc.2024.1277749

**Published:** 2024-01-23

**Authors:** Mizuki Tagami, Hiroaki Kasashima, Anna Kakehashi, Atsuko Yoshikawa, Mizuho Nishio, Norihiko Misawa, Atsushi Sakai, Hideki Wanibuchi, Masakazu Yashiro, Atsushi Azumi, Shigeru Honda

**Affiliations:** ^1^ Department of Ophthalmology and Visual Sciences, Graduate School of Medicine, Osaka Metropolitan University, Osaka, Japan; ^2^ Ophthalmology Department and Eye Center, Kobe Kaisei Hospital, Kobe, Japan; ^3^ Molecular Oncology and Therapeutics, Osaka Metropolitan University Graduate School of Medicine, Osaka, Japan; ^4^ Department of Molecular Pathology, Graduate School of Medicine, Osaka Metropolitan University, Osaka, Japan; ^5^ Department of Radiology, Kobe University Graduate School of Medicine, Kobe, Japan

**Keywords:** ocular adnexa MALT lymphoma, extranodal marginal zone lymphoma of mucosa-associated lymphoid tissue type, RNA sequencing, epithelial-mesenchymal transition, vimentin, tumor stroma, artificial segmentation image

## Abstract

**Purpose:**

To examine the molecular biological differences between conjunctival mucosa-associated lymphoid tissue (MALT) lymphoma and orbital MALT lymphoma in ocular adnexa lymphoma.

**Methods:**

Observational case series. A total of 129 consecutive, randomized cases of ocular adnexa MALT lymphoma diagnosed histopathologically between 2008 and 2020.Total RNA was extracted from formalin-fixed paraffin-embedded tissue from ocular adnexa MALT lymphoma, and RNA-sequencing was performed. Orbital MALT lymphoma gene expression was compared with that of conjunctival MALT lymphoma. Gene set (GS) analysis detecting for gene set cluster was performed in RNA-sequence. Related proteins were further examined by immunohistochemical staining. In addition, artificial segmentation image used to count stromal area in HE images.

**Results:**

GS analysis showed differences in expression in 29 GS types in primary orbital MALT lymphoma (N=5,5, FDR q-value <0.25). The GS with the greatest difference in expression was the GS of epithelial-mesenchymal transition (EMT). Based on this GS change, immunohistochemical staining was added using E-cadherin as an epithelial marker and vimentin as a mesenchymal marker for EMT. There was significant staining of vimentin in orbital lymphoma (*P*<0.01, N=129) and of E-cadherin in conjunctival lesions (*P*=0.023, N=129). Vimentin staining correlated with Ann Arbor staging (1 versus >1) independent of age and sex on multivariate analysis (*P*=0.004). Stroma area in tumor were significant difference(*P*<0.01).

**Conclusion:**

GS changes including EMT and stromal area in tumor were used to demonstrate the molecular biological differences between conjunctival MALT lymphoma and orbital MALT lymphoma in ocular adnexa lymphomas.

## Introduction

Lymphomas are a group of ocular adnexa malignant tumors that arise as clonal expansions of B lymphocytes, T lymphocytes, or natural killer (NK) cells (1). In fact, lymphoma is the most frequent neoplasm in the ophthalmological area (2, 3). The most common lymphoma subtypes of the orbital and ocular conjunctiva are of B-cell origin and include mucosa-associated lymphoid tissue (MALT) type, follicular lymphoma (FL), diffuse large B-cell lymphoma (DLBCL), and mantle cell lymphoma (MCL) (2–4).

For MALT lymphoma, one of the indolent B-cell lymphomas, the incidence has been reported to be higher in East Asian populations as compared to Western countries, and this may be the result of genetic or environmental factors (5, 6). In Japan, MALT lymphoma is the most common primary orbital tumor diagnosed, with this trend potentially continuing given the recent age structure that is associated with aging in Japan (7). In addition, as compared to IgG4 related orbital disease, there was significant up-regulation of some of the genes observed, including matrix metallopeptidase 12 (MMP12) and secreted phosphoprotein 1 (SPP1) (8).

Conjunctival MALT lymphomas are generally considered to have a good prognosis as compared to orbital MALT lymphoma. These are likely to be classified as I and II in the AJCC T stage, although their prognosis may depend on the genetic background that is present (5, 9, 10). In recent years, there have been studies that have investigated to gene expression differences wtih intersection mRNAs were involved in the activation of some cancer-related pathways, including PI3K/AKT, Ras, JAK-STAT, and NF-kappa B signaling pathway. PDGFRA, CXCL12, and CCL19 were the most significant central genes in the signal-net analysis. in some kinds of lymphomas (8, 11).

In other hands. In our study of Hematoxylin-Eosin(HE) staining slides of MALT lymphoma using machine learning, we reported that the two groups of lymphomas can be morphologically differentiated using machine learning at a rate of over 80% (12). This previous study also suggested that the two groups of MALT lymphomas may have different characters.

In the present study, we examined the differences in gene expression clusters between conjunctival MALT and orbital MALT lymphomas using the RNA sequencing method, with the biological differences between the two types of ocular adnexa MALT lymphoma then investigated.

## Materials and methods

### Selection of cases and collation of clinicopathological data

This study was a retrospective, observational, case series. Institutional Review Board (IRB)/Ethics Committee approval was obtained (approval No.4236), and the described research adhered to the tenets of the Declaration of Helsinki. Written, informed consent was obtained from all patients before enrollment. Between April 2008 and April 2020, a total of 129 patients were treated by ophthalmologists (AA, MT) and from whom we were able to formalin-fixed paraffin-embedded (FFPE)blocks with residual MALT lymphomas, which were subsequently identified. The diagnoses of conjunctival MALT lymphoma and orbital MALT lymphoma were based on clinical, radiographic (computed tomography (CT), magnetic resonance imaging (MRI)), histological and flow cytometric studies, and molecular genetic analyses, such as gene rearrangement.

Histopathologic examination of the tumor specimens was performed, and included staining with hematoxylin-eosin (HE) and immunohistochemical analyses. The following panel for B-cell lymphomas is currently recommended: CD3, CD5, CD10, CD20, and κ and λ light chains. All MALT lymphomas in the present study were classified according to the 2017, 4th edition of the WHO classification (1).

In addition, in this case study, immunoglobulin JH rearrangement was positive in all cases by Southern blotting using fresh specimens for the diagnosis of monoclonality of lymphoma.

The clinical data collected included age, sex, symptoms, clinical findings, systemic involvement according to the Ann Arbor staging classification and to the American Joint Committee on Cancer (AJCC) TNM classification system (9). In some patients, not all of the clinical data were available. The diagnostic systemic work-up of ocular MALT lymphoma usually included CT, full-body positron emission tomography-computed tomography (PET-CT), or MRI.

### RNA sequencing

#### RNA extraction and analysis

Total RNA was extracted from FFPE tissue blocks using NucleoSpin total RNA FFPE XS kit for RNA from FFPE (Macherey-Nagel GmbH & Co. KG, Duren, Germany) and purified by the RNeasy Mini Kit (QIAGEN, Hilden, Germany), followed by DNase treatment. After quantification using a Nanodrop 1000 spectrophotometer (Thermo Fisher Scientific, Tokyo, Japan), RNA was processed for RNA sequencing using random primers utilizing the SMART (switching mechanism at the 5′ end of the RNA template) methods. Poly(A) RNA was isolated using the NEBNext^®^ Poly(A) mRNA Magnetic Isolation Module, and barcoded libraries were made using the AMPure XP Kit (Beckman Coulter, Inc., CA). Libraries were pooled on the SMART-seq Stranded Kit (Takara Bio, Tokyo,Japan). Sequencing FastQ files were uploaded to BaseSpace and processed with RNA-Seq Alignment App (Illumina) to obtain raw read counts for each gene.

#### Bioinformatics analysis

Gene set enrichment analysis (GSEA) was performed using Gene Pattern, GSEA 20.2.4 (https://cloud.genepattern.org/gp/pages/index.jsf) with 1000 gene-set permutations using the gene-ranking metric T-test with the collections h.all.v7.4.symbols (Hallmarks).

### Immunohistochemistry (IHC)

The HE staining protocol was performed according to the standard laboratory protocol. Immunohistochemical expression analyses were performed on 3-μm-thick FFPE tissue sections using the following antibodies: anti-human vimentin mouse monoclonal (1:50, clone: V9; #ab8069; Abcam, Cambridge, UK), anti-human E-cadherin mouse monoclonal (1:50, clone: 36/E-Cadherin; BD Bioscience, NJ), anti-human ZEB1 rabbit monoclonal (1:50, clone:EPR17375;ab203829; Abcam, Cambridge, UK), anti-human Twist mouse monoclonal (1:50, clone:10E4E6;ab175430; Abcam, Cambridge, UK), Universal Elite ABC kit (PK-6200; Vector Laboratories, Burlingame, CA), Elite ABC Mouse kit (PK-6102; Vector Laboratories, Burlingame, CA), and ABC-AP Mouse IgG kit (AK-5002; Vector Laboratories). Tissue sections were incubated in ImmPACT DAB (Vector Laboratories) until the desired staining intensity developed. The sections were then counterstained with hematoxylin and mounted. Stained sections were viewed under an Olympus BX53+DP74 microscope.

### Image analysis

Tissues immunostained for vimentin and E-cadherin were evaluated in a blinded manner by two specialists (MT and AK). The first field for evaluation in each tumor lesion was selected randomly, and subsequently, 10 fields were systematically examined at 400× magnification using a mesh.

Vimentin and E-cadherin expressions were analyzed visually as the presence or absence of cell staining, with the samples divided semi-quantitatively into groups based on a score of 0 to 3 (0, none: 0-1/field; 1, weak: 1-<5/field; 2, strong: 5-<10/field; 3, very strong ≥10/field).

In addition, the HE images of conjunctival MALT and orbital MALT lymphomas were analyzed fully automatically. Randomly-selected image patches with an image resolution of 1600x1200 were extracted from the HE images. Then, the stroma area was segmented on the image patch and the number of pixels in the stroma area was counted. This model reproduces a wide range of colors by mixing the three primary colors of red (R), green (G), and blue (B). In this color model, each pixel consists of values of R, G, and B. Frequently, color images are represented by the RGB color model. Because the stroma area is mainly pink in color, our dedicated software automatically segmented and counted the pixels which met the following conditions for the pixel value in the RGB color space; (i) the R-value of the pixel was higher than 200, (ii) the G-value was between 175 and 225, and (iii) the B-value was lower than that of the R-value. RGB color model is a type of color representation method.

The pixel counts of the stroma area were compared between conjunctival MALT and orbital MALT lymphomas. This figure shows a simple flowchart for this automatic determination. (**Supplementary Figure 1**).

Our source code for the automatic analysis of HE images is available at the following https://github.com/jurader/automatic_analysis_of_MALT_HE_image/tree/main (13).

For demo, please click “Open in Colab” button in the notebook, https://github.com/jurader/automatic_analysis_of_MALT_HE_image/blob/main/Automatic_analysis_of_MALT_HE_image.ipynb.

### Statistical analyses

Clinical and histopathological characteristics are summarized using descriptive statistics. Correlations between immunohistochemical, demographic, and clinicopathological factor data were assessed using the *t*-test and the chi-squared test. IHC scores were assessed by the Mann-Whitney U test. Interobserver agreement was assessed using the κ statistic for the two pathologist raters (14), with: κ <0 indicating no agreement; κ = 0.0 to 0.19, poor; κ = 0.20 to 0.39, fair; κ = 0.40 to 0.59, moderate; κ = 0.60 to 0.79, substantial; and κ = 0.80 to 1.0, almost perfect agreement (15). For the multivariate analysis, odds ratios and 95% confidence intervals (CIs) were calculated by logistic regression analysis. Statistical analyses were performed using SPSS Statistics software, version 22 (IBM Japan, Tokyo, Japan). *p <*0.05 was considered significant.

## Results

### Clinical findings

**Table 1** summarizes the clinical findings of the cohort. All 129 patients (100%) were East Asian, with 49 men and 80 women, and a mean age at presentation of 65 ± 17 years. There was a significant difference in the age between the conjunctival cases and the orbital cases (*P*=0.0000054). Fifty-three patients (40%) had conjunctival MALT lymphoma, while 76 (60%) had orbital MALT lymphoma. The Ann Arbor staging designations at the time of presentation in conjunctival MALT lymphoma were: IE (87%, n = 46), II (3%, n = 2), III (0%, n = 0), and IV (2%, n = 1). In orbital MALT lymphoma, they were: IE (68%, n = 52), II (20%, n = 16), III (4%, n = 3), and IV (4%, n = 3).

**Table 1:** Clinical findings in ocular MALT lymphoma.

N (%) of Patients Ocular MALT lymphoma				
Ocular MALT lymphoma				
	ALL	conjunctival lymphoma	orbital lymphoma	*p* value
Total	129(100)	53(40)	76(60)	
SEX				
MALE	49(37)	18(34)	31(40)	
Female	80(63)	35(66)	45(60)	N,S.
Age at persentation				
y(average)	65±17	60±21	70±13	*p*<0.01*
Laterarcy				
unilateral	124(97)	51(96)	73(96)	
bilateral	5(3)	2(49	3(4)	N,S.
Ann Arbor stage				
IE	98(76)	46(87)	52(68)	
II	18(13)	2(3)	16(20)	
III	3(2)	0(0)	3(4)	
IV	4(3)	1(2)	3(4)	
N/A	6(4)	4(8)	2(4)	
AJCC T Stage				
T1	49 ( 37)	49(98)	0(0)	
T2	68(55)	0(0)	68(90)	
T3	3(2)	0(0)	3(4)	
T4	3(29	1(2)	3(4)	
N/A	6(4)	4(8)	2(4)	

MALT, mucosa-associated lymphoid tissue; AJCC, American Joint Committee on Cancer; N/A, no answer; N.S., no significant changes. Values of p <0.05 were considered significant; * = significant change.

The AJCC 8th edition T designations at the time of presentation were T1 (92%, n = 49), T2 (0%, n = 0), T3 (0%, n = 0), and T4 (2%, n = 1) in conjunctival MALT lymphoma, while they were T1 (0%, n = 0), T2 (89%, n = 68), T3 (3%, n = 3), and T4 (3%, n = 3) in orbital MALT lymphoma (**Table 1**). In addition, bilateral conjunctival lesions were classified as stage II by the Ann Arbor classification. In principle, as the lesions that extended beyond the orbital septum were classified as orbital lymphoma, the AJCC stage 2 was 0 in this retrospective study.

### RNA sequencing

**Figure 1:**
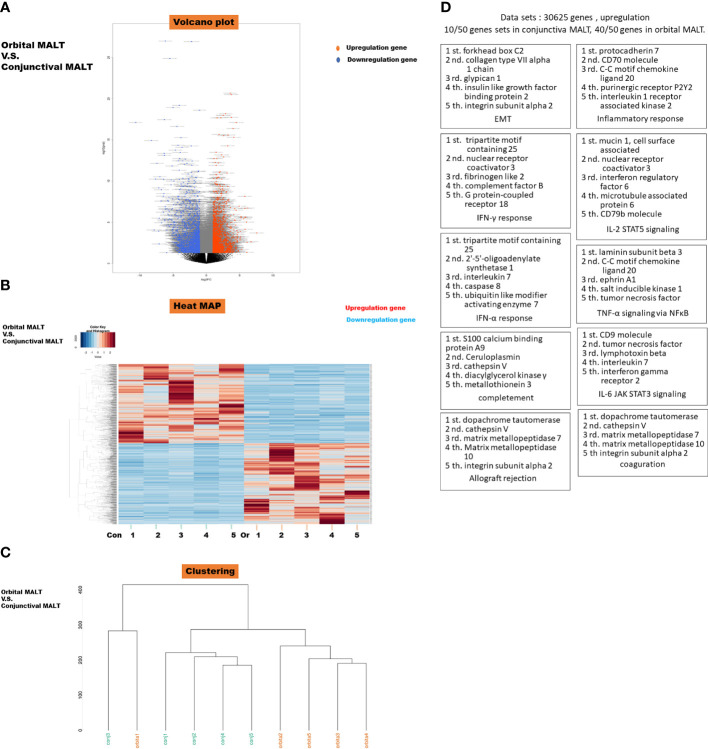
**(A)** Volcano plot of messenger RNAs (mRNAs). Blue dots, mRNA down-regulation; red dots, up-regulation; black dots, nonsignificant expression. Horizontal axis, fold change (FC); vertical axis: p value. **(B)** Heatmap obtained from unsupervised hierarchical clustering analysis using differentially expressed genes. The red to blue spectrum corresponds to high to low values. Con: conjunctival MALT, Or: orbital MALT **(C)** Clustering MAP. **(D)** Top 10 Gene sets in Gene set enrichment analysis (GSEA) were upregulated in orbital MALT lymphoma compared with conjunctival MALT lymphoma. This schema show each cluster represents an increased gene of TOP5.

All stages of conjunctival MALT are stage I(n=5), 3 cases of orbital MALT stage I, 1 case of stage II, and 1 case of stage III(n=5). All FFPE slides from which RNA was extracted had tumor content of over 80%. Results of RNA sequencing for orbital MALT lymphoma compared with conjunctival MALT lymphoma in volcano blot (**Figure 1A**). Heatmap and clustering map obtained from unsupervised hierarchical clustering analysis using differentially expressed genes. (**Figures 1B, C**).

The dataset had 30625 features (genes). 10/50 gene sets were upregulated in phenotype conjunctival MALT lymphoma and 40/50 gene sets are upregulated in phenotype orbital lymphoma (**Figure 1D**). Details of the specific 200 EMT gene sets are presented in **Supplementary Table 1**.

**Figure 2:**
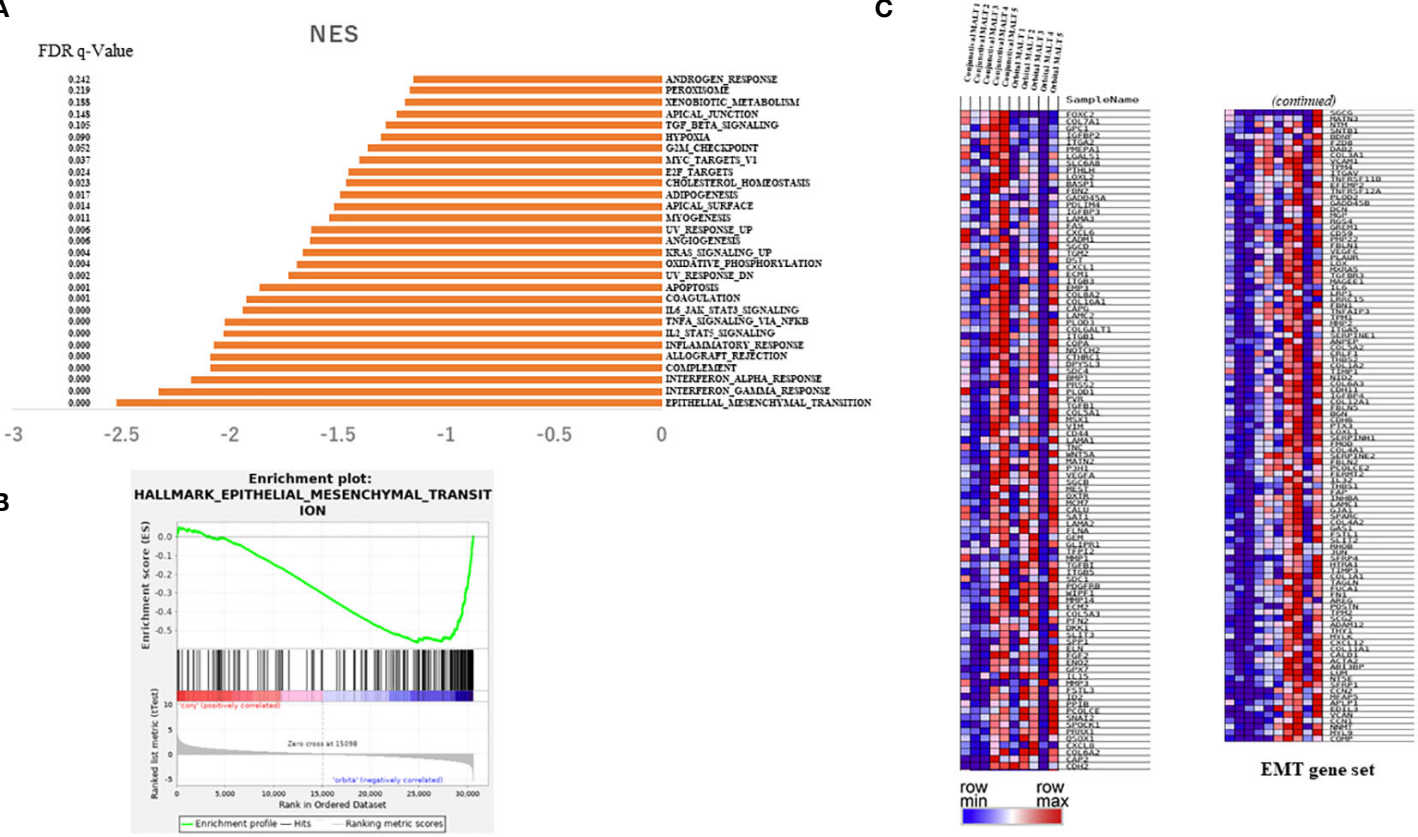
Results of RNA sequencing for the tissue of conjunctival MALT lymphoma compared with orbital MALT lymphoma. **(A)** Histogram showing pathways enriched in orbital MALT lymphoma. **(B)** GSEA plots of enrichment in conjunctival MALT lymphoma and orbital MALT lymphoma (n = 5). **(C)** Heatmap obtained from unsupervised hierarchical clustering analysis using differentially expressed genes in the EMT pathway. The red to blue spectrum corresponds to the high to low values. MALT, mucosa-associated lymph tissue; GSEA, gene set enrichment analysis; EMT, epithelial-mesenchymal transition.

To investigate the difference in clinical outcomes, we performed transcriptomic profiling of orbital MALT lymphoma (n = 5) and conjunctival MALT lymphoma (n = 5). GSEA analysis showed that there were 22 gene sets in the collections h.all.v7.4.symbols (Hallmarks) that were upregulated in orbital MALT lymphoma (FDR q-value<0.05), including epithelial-mesenchymal transition (EMT), inflammatory response, and IL6-JAK-STAT3 signaling, in addition to 29 gene sets when the FDR q-value was <0.25 (**Figure 2A**). Of these, enrichment of the EMT pathway was the top-ranked signature (normalized enrichment score (NES) = -2.51) (**Figures 2B, C**).

In EMT associated genes including TGF-beta, SNAIL, ZEB1, twist and ZEB1, mRNA expressions of RNA sequence compared conjunctival MALT with orbital MALT in EMT associated genes. (n=5.5). As result, there were significant change. (**Supplementary Figure 2**).

### Histological results with HE and immunohistochemical staining

**Figure 3:**
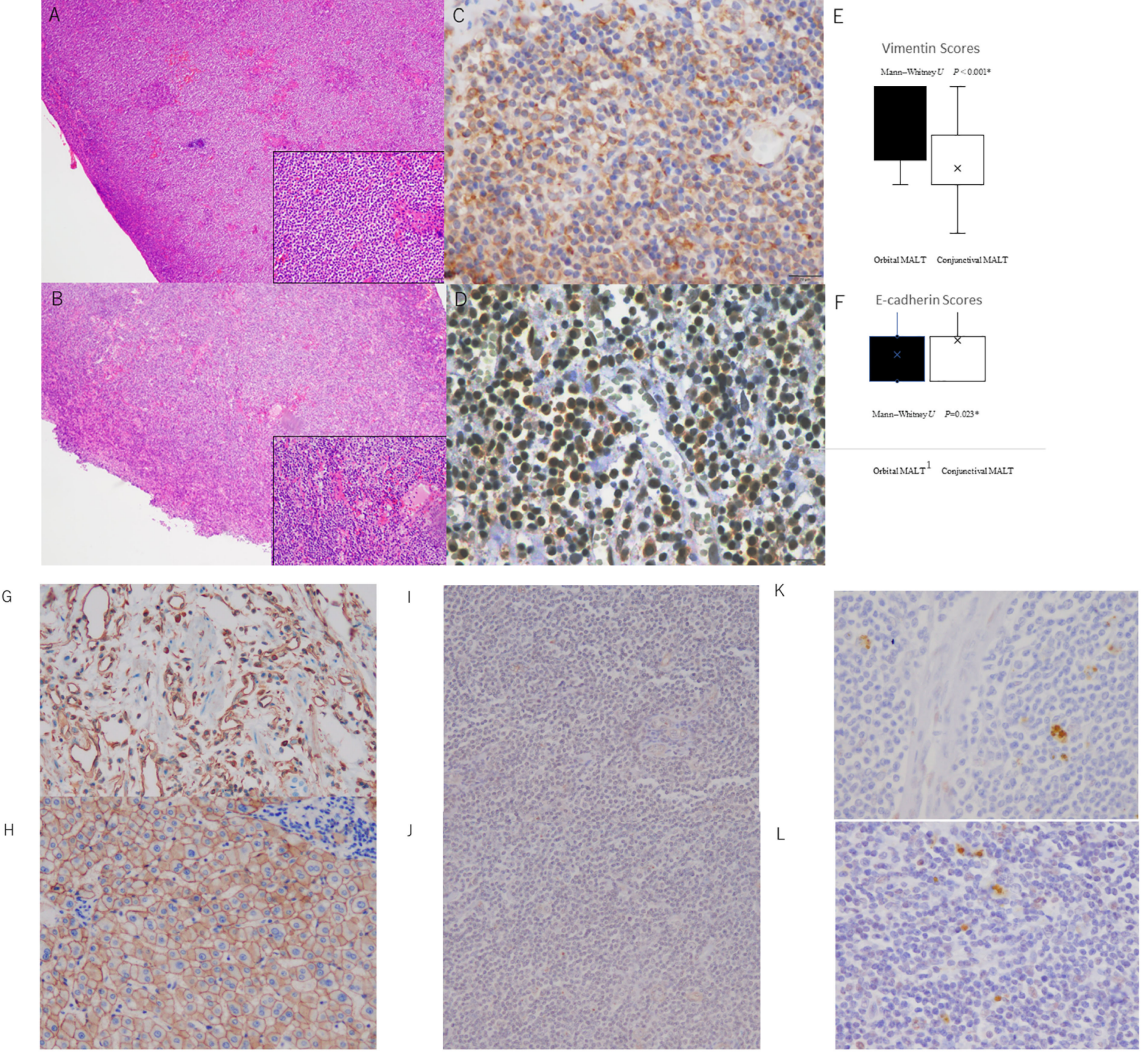
**(A)** Photomicrographs showing hematoxylin and eosin staining (HE) and immunohistochemical staining of conjunctival MALT lymphoma and orbital MALT lymphoma with vimentin and E-cadherin antibodies. **(A)** Conjunctival MALT lymphoma showing uniform small lymphoma cells. The bottom right inset shows a higher power of HE (original magnification, ×4; inset: original magnification ×20). **(B)** Orbital MALT lymphoma showing uniform small lymphoma cells with fibrotic stroma area. The bottom right inset shows a higher power of HE (original magnification, ×4; inset: original magnification ×20). **(C)** Orbital MALT lymphoma showing strong vimentin immunostaining with tumor lymphoma cells and tumor stromal area (original magnification, ×40). **(D)** Conjunctival MALT lymphoma showing E-cadherin immunostaining with tumor lymphoma cells and tumor cytoplasm (original magnification, ×40). **(E)** Vimentin IHC scores in orbital MALT lymphoma express a high s score (score: 2.18) compared with conjunctival MALT lymphoma (score: 1.42) (*P*=0.0000012) (upper). **(F)** E-cadherin IHC scores in conjunctival MALT lymphoma express a high s score (score: 1.14) compared with orbital MALT lymphoma (score: 0.59) (*p* = 0.023) (bottom). B. Control slide Photomicrographs. **(G)** Positive control for Vimentin (original magnification, ×20). **(H)** Positive control for E-cadherin (original magnification, ×20). **(I)** Negative control for Vimentin (original magnification, ×20). **(J)** Negativecontrol for E-cadherin (original magnification, ×20). **(K)** Orbital MALT lymphoma showing strong ZEB1 immunostaining with tumor lymphoma cells and tumor stromal area (original magnification, ×40). **(L)** Orbital MALT lymphoma showing strong Twist immunostaining (original magnification, ×40).

HE staining was used to evaluate the fibrosis score in the tumor. As shown in **Figure 3A**, conjunctival MALT lymphoma samples with no fibrosis (**Figure 3AA**) exhibited normal uniform lymphoma cells with small fibrotic cords. Orbital MALT lymphoma samples with mild or severe fibrosis exhibited disordered architecture and bridging fibrosis (**Figure 3AB**).

Scores for vimentin immunohistochemical staining were 2.18 ± 0.80 in orbital MALT lymphoma (n = 76) and 1.42 ± 0.58 in conjunctival MALT lymphoma (n = 53; *p*=0.0000012 (**Figure 3AC**). Scores for E-cadherin immunohistochemical staining were 0.59 ± 0.78 in orbital MALT lymphoma (n = 76) and 1.14 ± 0.80 in conjunctival MALT lymphoma (n = 53; *p* = 0.023) (**Figure 3AD**). The total vimentin staining agreement was substantial (κ = 0.64). The agreement for the E-cadherin staining category was moderate (κ = 0.51) (**Figures 3AE, AF**). There were positive and negative controls in this experiment in **Figure 3B** (**Figures 3BG–I**). In **Figure 3C**, investigation of EMT transcriptional factors, there were ZEB-1 staining (**Figure 3CJ**) and Twist staining (**Figure 3CK**). Scores for ZEB-1immunohistochemical staining were 1.0 ± 0.77 in orbital MALT lymphoma and 0.70± 0.45 in conjunctival MALT lymphoma (*p* = 0.33). Scores for TWIST immunohistochemical staining were 1.4 ± 0.48 in orbital MALT lymphoma and 1.3± 0.64 in conjunctival MALT lymphoma (*p* = 0.71).

### Image analysis for stroma area in HE images

**Figure 4:**
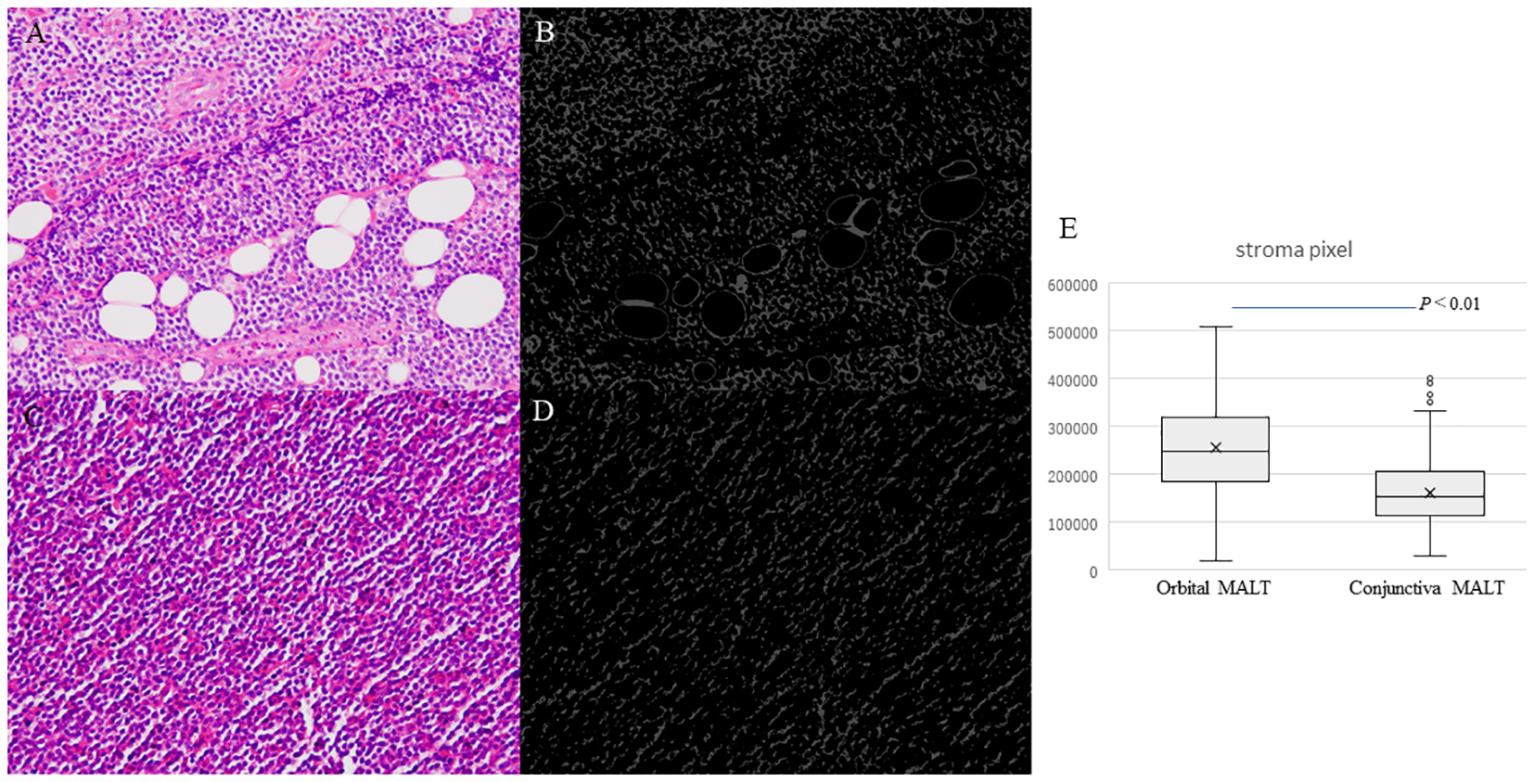
Photomicrographs showing hematoxylin and eosin staining (HE) for counting stromal area. **(A)** Orbital MALT lymphoma HE image showing fibrotic stroma and fat tissue. **(B)** Segmentation result for counting stromal area in orbital MALT. **(C)** Conjunctival MALT lymphoma HE image showing a few stromal area. **(D)** Segmentation result for counting stroma area in conjunctival MALT. **(E)** Boxplots comparing conjunctival MALT and orbital MALT for the number of the stroma area (*P*<0.01).

Artificial segmentation images for the pixel value in the RGB color space were converted from the HE images obtained from 53 cases of conjunctival MALT and 74 cases of orbital MALT to the image patches (n=127, total number of image patches=1270) (**Figures 4A–D**). Two cases were excluded from this study due to image problems.

Boxplots comparing conjunctival MALT and orbital MALT show that the number of the pixel counts in the stroma area was significantly different between the two groups (**Figure 4E**. P<0.01).

#### Correlation of EMT-associated IHC with Ann Arbor classification

Vimentin scores were correlated with systemic involvement (Ann Arbor staging I versus >I) and independent of age and sex as determined by the multivariate analysis using logistic regression analysis (*p* = 0.004, n = 129) (**Table 2**).

**Table 2:** Multivariate analysis between Ann Arbor staging at diagnosis (I or >I) and various clinicopathological and molecular factors.

Multivariate analysis				
Variable	n = 129	Odds	95% CI	*P*
**Age**	65 ± 17 years	1.024	0.992-1.056	0.140
**Sex**	Male 49, Female 91	0.526	0.185-1.494	0.228
**Vimentin IHC Scores**	Conjunctival: 1.42 Orbital: 2.18	**2.448**	**1.341-4.476**	0.004*
**E-cadherin IHC Scores**	Conjunctival: 1.14 Orbital: 0.59	0.286	0.764-2.488	0.286

*P<0.05 logistic regression analysis.

AJCC, American Joint Committee on Cancer; CI, confidence interval; HR, hazard ratio.

Values of *p <*0.05 were considered significant; * = significant change.

## Discussion

In the 10 cases examined in this study, many differences in gene expression clusters were observed between conjunctival MALT lymphoma and orbital MALT lymphoma for the RNA sequencing and bioinformatics analysis. The differential expression of the genes and pathway analysis with GSEA detected by RNA sequencing for conjunctival MALT lymphoma versus orbital MALT lymphoma may provide insight into the genetic background of these two different MALT lymphoma lesions.

GSEA analysis in orbital MALT lymphoma identified 29 pathways that exhibited significant differences as compared to that observed with conjunctival MALT lymphoma: EMT; interferon-gamma response; interferon-alfa response; complement; allograft rejection; inflammatory response; IL-2-STAT5-signaling; tumor necrosis factor-alfa (TNF-α) signaling via nuclear factor-kappaB (NF-κB); IL-6-Janus kinase (JAK)-STAT3-signaling; coagulation; apoptosis; ultraviolet (UV) response down; oxidative phosphorylation; KRAS signaling up; angiogenesis; UV response up; myogenesis; apical surface; adipogenesis; cholesterol homeostasis; E2F targets; MYC targets; G2M check point; hypoxia; transforming growth factor beta (TGF-β) signaling; apical junction; xenobiotic metabolism; peroxisome; and androgen response.

Of these and especially for EMT, immunohistochemical staining was performed for E-cadherin, which is an epithelial marker, and for vimentin, which is a mesenchymal marker (16). Among them, the cluster that showed the largest change at the RNA level was the EMT cluster, and we decided to investigate this further in this study.

IN addition, this switch in the cell differentiation is mediated by key transcription factors, which include SNAIL, ZEB1, twist and TGF-β, and the functions of these are finely regulated at the transcriptional, translational, and post-translational levels.

The EMT process results in the downregulation of epithelial and the activation of the mesenchymal cell characteristics, with these changes contributing pathologically to the fibrosis and cancer progression (17). This switch in the cell differentiation is mediated by key transcription factors, which include SNAIL, ZEB1, Twist and TGF-β, and the functions of these are finely regulated at the transcriptional, translational, and post-translational levels (18).

In lymphoma, there have been reports of biological activity and prognosis in patients with DLBCL and MCL and in an *in vitro* study (19). Furthermore, EMT transcription factors (EMT-TFs) include SNAIL, ZEB1, twist and ZEB1 during normal blood cell development have emerged, which appear to be largely independent of classical EMT processes. EMT-TFs have also begun to be implicated in the development and pathogenesis of malignant hematological diseases such as leukemia and lymphoma (20–24).

The results of the present study, showed that EMT may be a key molecular cluster of ocular MALT lymphoma invasion. It has been reported that the EMT pathway is strongly associated with cancer initiation and progression (25). On the other hand, conjunctival MALT may contain RNA from conjunctival epithelial components, and IHC may also lead to misleading staining.

Previous reports have also linked molecular and genetic backgrounds to the prognosis (26). In ocular MALT lymphoma, chromosomal abnormalities are associated with MALT lymphoma: trisomy 3, 7, 12, and 18, somatic deletion and/or mutation of A20, and the chromosomal translocations are especially found in patients with ocular MALT, and that genetic translocations affect or target different regulating genes, thereby leading to the formation or upregulation of proteins that ultimately activate NF-κB (27). In the present cluster analysis, the TNF-α signaling via NF-κB showed a significant difference in the pathway activation in orbital MALT lymphoma as compared to that observed with conjunctival MALT lymphoma.

Our multivariate analysis showed that the vimentin immunostaining was correlated with T1 versus >T1 T staging, and was a factor that was independent of sex and age, which corresponded to our RNA sequencing results. The reason why the Ann Arbor classification I versus >I is important is that, as mentioned in past reports, conjunctival and orbital MALT lymphomas have a very high therapeutic response to local radiation therapy alone, with the complication rate considered to be very acceptable. In other words, the patient’s treatment policy and quality of life can significantly change depending on the conditions, and whether or not it remains locally in the orbit or is at a more advanced stage (28). The data that we determined at the molecular level of the ocular adnexa have not been previously demonstrated. However, earlier articles have reported that vimentin staining was associated with treatment resistance in systemic lymphomas such as DLBCL (29–31).

These findings are in line with our hypothesis, that the origin of ocular adnexa lymphoma is initially the same, but can have a strong epithelial propensity (such as that observed with the strong E-cadherin staining) and thus, subcutaneously extend to the conjunctiva and become conjunctival MALT. As a result, a strong mesenchymal tendency (such as strong vimentin staining) may exist and subsequently expand and invade deep into the orbit. This may correlate with the TNM stage and Ann Arbor classification of the whole body.

In other hands, Asakage et al. described that gene expression of inflammation and fibrosis markers was increased in IgG4ROD cases, and in our present study, an increase in inflammation-related clusters was also observed in orbital MALT. The pathology of both MALT and igG4ORD may be explained by inflammation. These orbital diseases may fall on a large spectrum.

Based on these overall findings, it is our belief that a systemic work-up should be actively performed in these types of cases. In addition, in this study, we showed that there was a statistically significant difference in the stromal area in the tumor between the two ocular adnexa MALT lymphomas. Although not all of these relationships are clear, there is some agreement between gene expression and imaging results that may be relevant to clinical prognostic differences. In treatment, previous reported radiation therapy could be the recommended treatment for stage IEA stage patients, and systemic chemotherapy (rituximab and R-CHOP) be indicated in selected stage IIEA stage patients and in patients with stage IIIEA disease (32–34).

The limitations of the present study include its retrospective design and the relatively small number of cases that were collected from a single institution. In all cases, immunoglobulin JH rearrangement was positive by Southern blotting with fresh samples to diagnose monoclonality of lymphoma, but there were also false positives and false negatives, and the differential diagnosis was lymphoproliferative diseases including If IgG4-related. There is a possibility that cases of orbital disease (IgG4ROD) are mixed. In addition, Regarding the tumor stroma areas, artificial intelligence method was used, however it is difficult to completely eliminate the influence of artificial objects, and the influence of artificial cutting and embedding may be added to the tumor stroma. This report could have produced both a selection and confounding bias, and there were no cases that involved the eyelid previous report described association with poor prognosis (35).

In conclusion, to the best of our knowledge, orbital MALT lymphoma exhibits 29 different gene sets on GSEA analysis as compared to that found for conjunctival MALT lymphoma on RNA sequencing. Thus, the EMT pathway could be a critical clue for understanding the molecular biological and clinical differences between conjunctival MALT lymphoma and orbital MALT lymphoma. In addition, There were significant differences in the stromal area in the tumor between the two ocular adnexa MALT lymphomas. However, the representative EMT-TFs that we searched for this time, such as ZEB-1 and TWIST, did not show any significant difference between the two groups, and the detailed pathway requires further investigation in the future.

## Data availability statement

The original contributions presented in the study are included in the article/**Supplementary Material**, further inquiries can be directed to the corresponding author.

## Ethics statement

The studies involving humans were approved by Osaka Metropolitan University Institutional Review Board (IRB)/Ethics Committee approval was obtained (approval No. 4236). The studies were conducted in accordance with the local legislation and institutional requirements. Written informed consent for participation in this study was provided by the participants’ legal guardians/next of kin.

## Author contributions

MT: Conceptualization, Data curation, Formal Analysis, Funding acquisition, Investigation, Methodology, Project administration, Resources, Visualization, Writing – original draft. HK: Data curation, Formal Analysis, Investigation, Methodology, Writing – original draft. AK: Formal Analysis, Supervision, Validation, Visualization, Writing – review & editing. AY: Formal Analysis, Methodology, Writing – original draft. MN: Data curation, Formal Analysis, Investigation, Methodology, Software, Writing – original draft. NM: Investigation, Writing – original draft. AS: Investigation, Writing – original draft. HW: Supervision, Writing – review & editing. MY: Supervision, Writing – review & editing. AA: Conceptualization, Supervision, Validation, Writing – review & editing. SH: Funding acquisition, Supervision, Validation, Visualization, Writing – review & editing.

## Supplementary material

Supplementary Figure 1 Detail for shows a simple flowchart for this automatic determination.

Supplementary Figure 2 mRNA expression of RNA sequence compared conjunctival MALT with orbital MALT in EMT associated genes. (n=5.5). N.S>:no significant change, * *p*<0.05.

Supplementary Table 1 Gene set enrichment analysis data.
