# Supplementary figures and images for: Stromal area differences with epithelial-mesenchymal transition gene changes in conjunctival and orbital mucosa-associated lymphoid tissue lymphoma
Source: Front Oncol. 2024 Jan 23;14:1277749. doi: 10.3389/fonc.2024.1277749 (PMC10845137; doi:10.3389/fonc.2024.1277749)

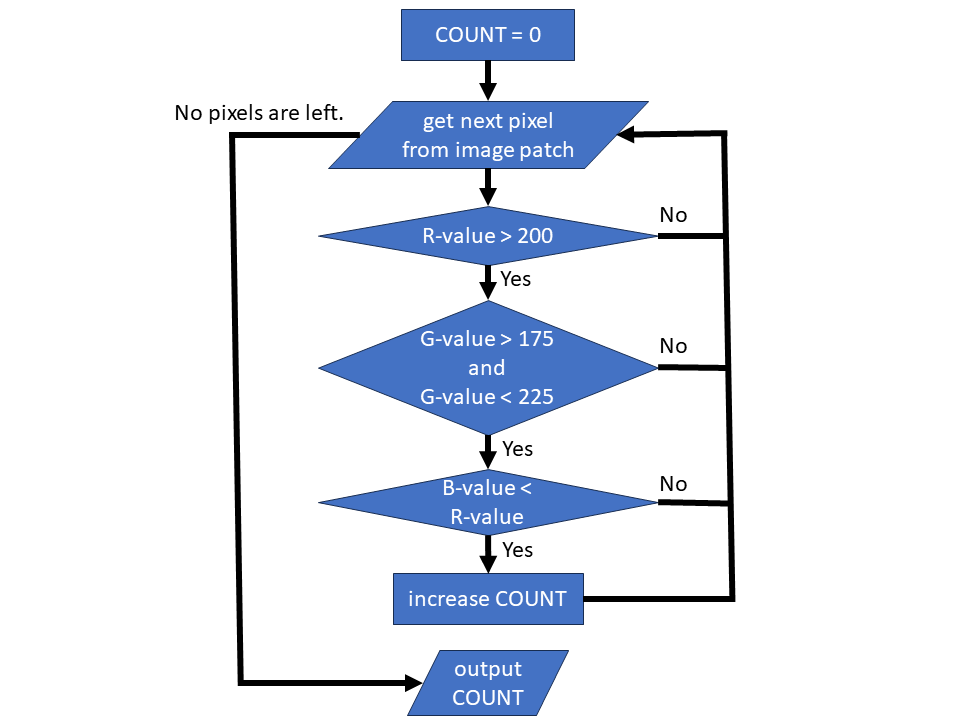

Supplement: Supplementary Figure 1 — Detail for shows a simple flowchart for this automatic determination. [file Image_1.tif]

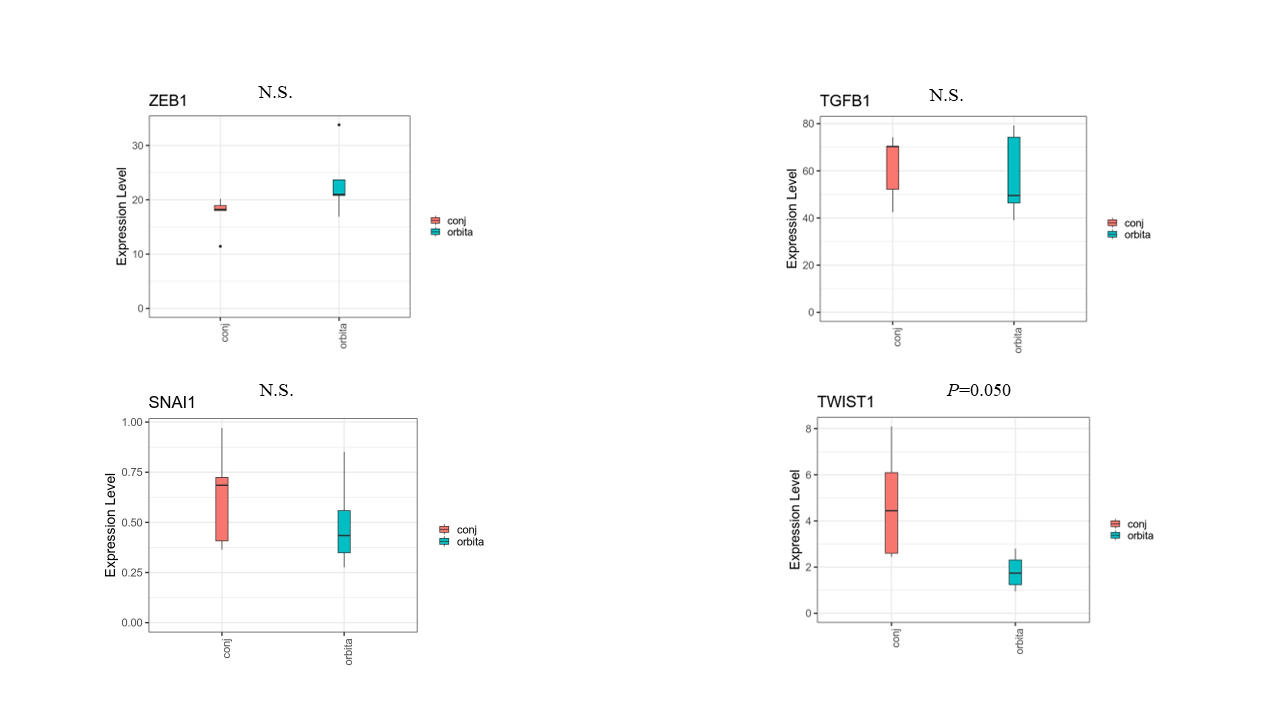

Supplement: Supplementary Figure 2 — mRNA expression of RNA sequence compared conjunctival MALT with orbital MALT in EMT associated genes. (n=5.5). N.S>:no significant change, * p<0.05. [file Image_2.tif]
