# Supplementary material for: Stromal area differences with epithelial-mesenchymal transition gene changes in conjunctival and orbital mucosa-associated lymphoid tissue lymphoma
Source: Front Oncol. 2024 Jan 23;14:1277749. doi: 10.3389/fonc.2024.1277749 (PMC10845137; doi:10.3389/fonc.2024.1277749)
Supplement: Supplementary Table 1 — Gene set enrichment analysis data. [file DataSheet_1.pdf]

Supplemental Table GSEA details.

	SYMBOL	TITLE	RANK IN GENE LIST		RUNNING ES	
1	FOXC2	forkhead box C2 [Source:HGNC Symbol;Acc:HGNC:3801]	36	4.025	0.0158	No
2	COL7A1	collagen type VII alpha 1 chain [Source:HGNC Symbol;Acc:HGNC:2214]	70	3.698	0.0304	No
3	GPC1	glypican 1 [Source:HGNC Symbol;Acc:HGNC:4449]	202	2.962	0.0386	No
4	IGFBP2	insulin like growth factor binding protein 2 [Source:HGNC Symbol;Acc:HGNC:5471]	235	2.844	0.0495	No
5	ITGA2	integrin subunit alpha 2 [Source:HGNC Symbol;Acc:HGNC:6137]	574	2.186	0.0477	No
6	PMEPA1	prostate transmembrane protein, androgen induced 1 [Source:HGNC Symbol;Acc:HGNC:14107]	1139	1.708	0.0363	No
7	LGALS1	galectin 1 [Source:HGNC Symbol;Acc:HGNC:6561]	1201	1.678	0.0414	No
8	SLC6A8	solute carrier family 6 member 8 [Source:HGNC Symbol;Acc:HGNC:11055]	1724	1.446	0.0304	No
9	PTHLH	parathyroid hormone like hormone [Source:HGNC Symbol;Acc:HGNC:9607]	1793	1.419	0.0341	No
10	LOXL2	lysyl oxidase like 2 [Source:HGNC Symbol;Acc:HGNC:6666]	2091	1.319	0.0299	No
11	BASP1	brain abundant membrane attached signal protein 1 [Source:HGNC Symbol;Acc:HGNC:957]	2349	1.248	0.0268	No

12	FBN2	fibrillin 2 [Source:HGNC Symbol;Acc:HGNC:3604]	3268	1.052	0.0010	No
13	GADD45A	growth arrest and DNA damage inducible alpha [Source:HGNC Symbol;Acc:HGNC:4095]	3275	1.050	0.0053	No
14	PDLIM4	PDZ and LIM domain 4 [Source:HGNC Symbol;Acc:HGNC:16501]	3741	0.971	-0.0059	No
15	IGFBP3	insulin like growth factor binding protein 3 [Source:HGNC Symbol;Acc:HGNC:5472]	3885	0.948	-0.0066	No
16	LAMA3	laminin subunit alpha 3 [Source:HGNC Symbol;Acc:HGNC:6483]	4102	0.918	-0.0098	No
17	FAS	Fas cell surface death receptor [Source:HGNC Symbol;Acc:HGNC:11920]	4237	0.899	-0.0104	No
18	CXCL6	C-X-C motif chemokine ligand 6 [Source:HGNC Symbol;Acc:HGNC:10643]	4338	0.885	-0.0100	No
19	CADM1	cell adhesion molecule 1 [Source:HGNC Symbol;Acc:HGNC:5951]	4401	0.877	-0.0083	No
20	SGCD	sarcoglycan delta [Source:HGNC Symbol;Acc:HGNC:10807]	4450	0.870	-0.0062	No
21	TGM2	transglutaminase 2 [Source:HGNC Symbol;Acc:HGNC:11778]	4504	0.861	-0.0043	No
22	DST	dystonin [Source:HGNC Symbol;Acc:HGNC:1090]	4657	0.841	-0.0057	No
23	CXCL1	C-X-C motif chemokine ligand 1 [Source:HGNC Symbol;Acc:HGNC:4602]	4695	0.837	-0.0034	No
24	ECM1	extracellular matrix protein 1 [Source:HGNC	4945	0.806	-0.0082	No

		Symbol;Acc:HGNC:3153]				
25	ITGB3	integrin subunit beta 3 [Source:HGNC Symbol;Acc:HGNC:6156]	5412	0.749	-0.0203	No
26	EMP3	epithelial membrane protein 3 [Source:HGNC Symbol;Acc:HGNC:3335]	5901	0.689	-0.0335	No
27	COL8A2	collagen type VIII alpha 2 chain [Source:HGNC Symbol;Acc:HGNC:2216]	6048	0.671	-0.0354	No
28	COL16A1	collagen type XVI alpha 1 chain [Source:HGNC Symbol;Acc:HGNC:2193]	6286	0.644	-0.0405	No
29	CAPG	capping actin protein, gelsolin like [Source:HGNC Symbol;Acc:HGNC:1474]	6440	0.627	-0.0429	No
30	LAMC2	laminin subunit gamma 2 [Source:HGNC Symbol;Acc:HGNC:6493]	6508	0.618	-0.0425	No
31	PLOD3	procollagen-lysine,2-oxoglutarate 5-dioxygenase 3 [Source:HGNC Symbol;Acc:HGNC:9083]	7336	0.527	-0.0674	No
32	COLGALT1	collagen beta(1-O)galactosyltransferase 1 [Source:HGNC Symbol;Acc:HGNC:26182]	8400	0.424	-0.1006	No
33	ITGB1	integrin subunit beta 1 [Source:HGNC Symbol;Acc:HGNC:6153]	8676	0.403	-0.1079	No
34	COPA	COPI coat complex subunit alpha [Source:HGNC Symbol;Acc:HGNC:2230]	8936	0.382	-0.1148	No
35	NOTCH2	notch receptor 2 [Source:HGNC Symbol;Acc:HGNC:7882]	9086	0.370	-0.1182	No
36	CTHRC1	collagen triple helix repeat containing 1	9385	0.347	-	No

		[Source:HGNC Symbol;Acc:HGNC:18831]			0.1265	
37	DPYSL3	dihydropyrimidinase like 3 [Source:HGNC Symbol;Acc:HGNC:3015]	10223	0.280	-0.1528	No
38	SDC4	syndecan 4 [Source:HGNC Symbol;Acc:HGNC:10661]	10409	0.266	-0.1578	No
39	BMP1	bone morphogenetic protein 1 [Source:HGNC Symbol;Acc:HGNC:1067]	10590	0.253	-0.1626	No
40	PRSS2	serine protease 2 [Source:HGNC Symbol;Acc:HGNC:9483]	10650	0.248	-0.1635	No
41	PLOD1	procollagen-lysine,2-oxoglutarate 5-dioxygenase 1 [Source:HGNC Symbol;Acc:HGNC:9081]	10830	0.236	-0.1684	No
42	PVR	PVR cell adhesion molecule [Source:HGNC Symbol;Acc:HGNC:9705]	10940	0.227	-0.1710	No
43	TGFB1	transforming growth factor beta 1 [Source:HGNC Symbol;Acc:HGNC:11766]	11591	0.185	-0.1916	No
44	COL5A1	collagen type V alpha 1 chain [Source:HGNC Symbol;Acc:HGNC:2209]	13432	0.075	-0.2518	No
45	MSX1	msh homeobox 1 [Source:HGNC Symbol;Acc:HGNC:7391]	14007	0.046	-0.2704	No
46	VIM	vimentin [Source:HGNC Symbol;Acc:HGNC:12692]	14008	0.046	-0.2702	No
47	CD44	CD44 molecule (Indian blood group) [Source:HGNC Symbol;Acc:HGNC:1681]	14010	0.046	-0.2701	No

48	LAMA1	laminin subunit alpha 1 [Source:HGNC Symbol;Acc:HGNC:6481]	14197	0.038	-0.2760	No
49	TNC	tenascin C [Source:HGNC Symbol;Acc:HGNC:5318]	14960	0.008	-0.3010	No
50	WNT5A	Wnt family member 5A [Source:HGNC Symbol;Acc:HGNC:12784]	15036	0.004	-0.3035	No
51	MATN2	matrilin 2 [Source:HGNC Symbol;Acc:HGNC:6908]	16534	-0.057	-0.3524	No
52	P3H1	prolyl 3-hydroxylase 1 [Source:HGNC Symbol;Acc:HGNC:19316]	16853	-0.072	-0.3626	No
53	VEGFA	vascular endothelial growth factor A [Source:HGNC Symbol;Acc:HGNC:12680]	17226	-0.093	-0.3744	No
54	SGCB	sarcoglycan beta [Source:HGNC Symbol;Acc:HGNC:10806]	17344	-0.100	-0.3779	No
55	MEST	mesoderm specific transcript [Source:HGNC Symbol;Acc:HGNC:7028]	18293	-0.152	-0.4084	No
56	OXTR	oxytocin receptor [Source:HGNC Symbol;Acc:HGNC:8529]	18362	-0.157	-0.4099	No
57	MCM7	minichromosome maintenance complex component 7 [Source:HGNC Symbol;Acc:HGNC:6950]	18432	-0.161	-0.4115	No
58	CALU	calumenin [Source:HGNC Symbol;Acc:HGNC:1458]	19092	-0.205	-0.4323	No
59	SAT1	spermidine/spermine N1-acetyltransferase 1 [Source:HGNC Symbol;Acc:HGNC:10540]	19114	-0.206	-0.4321	No
60	LAMA2	laminin subunit alpha 2 [Source:HGNC Symbol;Acc:HGNC:6482]	19465	-0.233	-0.4427	No

61	FLNA	filamin A [Source:HGNC Symbol;Acc:HGNC:3754]	19528	-0.239	-0.4437	No
62	GEM	GTP binding protein overexpressed in skeletal muscle [Source:HGNC Symbol;Acc:HGNC:4234]	19660	-0.248	-0.4469	No
63	GLIPR1	GLI pathogenesis related 1 [Source:HGNC Symbol;Acc:HGNC:17001]	20424	-0.307	-0.4707	No
64	TFPI2	tissue factor pathway inhibitor 2 [Source:HGNC Symbol;Acc:HGNC:11761]	20570	-0.318	-0.4742	No
65	MMP1	matrix metallopeptidase 1 [Source:HGNC Symbol;Acc:HGNC:7155]	20753	-0.332	-0.4787	No
66	TGFBI	transforming growth factor beta induced [Source:HGNC Symbol;Acc:HGNC:11771]	20800	-0.336	-0.4788	No
67	ITGB5	integrin subunit beta 5 [Source:HGNC Symbol;Acc:HGNC:6160]	20923	-0.346	-0.4814	No
68	SDC1	syndecan 1 [Source:HGNC Symbol;Acc:HGNC:10658]	21127	-0.361	-0.4865	No
69	PDGFRB	platelet derived growth factor receptor beta [Source:HGNC Symbol;Acc:HGNC:8804]	21624	-0.408	-0.5011	No
70	WIPF1	WAS/WASL interacting protein family member 1 [Source:HGNC Symbol;Acc:HGNC:12736]	21667	-0.411	-0.5007	No
71	MMP14	matrix metallopeptidase 14 [Source:HGNC Symbol;Acc:HGNC:7160]	21934	-0.436	-0.5076	No
72	ECM2	extracellular matrix protein 2	22159	-	-	No

		[Source:HGNC Symbol;Acc:HGNC:3154]		0.458	0.5131	
73	COL5A3	collagen type V alpha 3 chain [Source:HGNC Symbol;Acc:HGNC:14864]	22342	-0.475	-0.5170	No
74	PFN2	profilin 2 [Source:HGNC Symbol;Acc:HGNC:8882]	22429	-0.483	-0.5178	No
75	DKK1	dickkopf WNT signaling pathway inhibitor 1 [Source:HGNC Symbol;Acc:HGNC:2891]	22661	-0.505	-0.5233	No
76	SLIT3	slit guidance ligand 3 [Source:HGNC Symbol;Acc:HGNC:11087]	22697	-0.508	-0.5223	No
77	SPP1	secreted phosphoprotein 1 [Source:HGNC Symbol;Acc:HGNC:11255]	22709	-0.510	-0.5205	No
78	ELN	elastin [Source:HGNC Symbol;Acc:HGNC:3327]	22892	-0.528	-0.5243	No
79	FGF2	fibroblast growth factor 2 [Source:HGNC Symbol;Acc:HGNC:3676]	23043	-0.546	-0.5269	No
80	ENO2	enolase 2 [Source:HGNC Symbol;Acc:HGNC:3353]	23374	-0.578	-0.5353	No
81	GPX7	glutathione peroxidase 7 [Source:HGNC Symbol;Acc:HGNC:4559]	23494	-0.591	-0.5367	No
82	IL15	interleukin 15 [Source:HGNC Symbol;Acc:HGNC:5977]	23588	-0.602	-0.5372	No
83	MMP3	matrix metallopeptidase 3 [Source:HGNC Symbol;Acc:HGNC:7173]	23815	-0.627	-0.5420	No
84	FSTL3	follistatin like 3 [Source:HGNC Symbol;Acc:HGNC:3973]	23838	-0.629	-0.5401	No

85	ID2	inhibitor of DNA binding 2 [Source:HGNC Symbol;Acc:HGNC:5361]	24474	-0.701	-0.5580	No
86	PPIB	peptidylprolyl isomerase B [Source:HGNC Symbol;Acc:HGNC:9255]	24663	-0.725	-0.5611	No
87	PCOLCE	procollagen C-endopeptidase enhancer [Source:HGNC Symbol;Acc:HGNC:8738]	24742	-0.733	-0.5605	No
88	SNAI2	snail family transcriptional repressor 2 [Source:HGNC Symbol;Acc:HGNC:11094]	24919	-0.752	-0.5632	Yes
89	SPOCK1	SPARC (osteonectin), cwcv and kazal like domains proteoglycan 1 [Source:HGNC Symbol;Acc:HGNC:11251]	24929	-0.754	-0.5603	Yes
90	PRRX1	paired related homeobox 1 [Source:HGNC Symbol;Acc:HGNC:9142]	24941	-0.756	-0.5574	Yes
91	QSOX1	quiescin sulfhydryl oxidase 1 [Source:HGNC Symbol;Acc:HGNC:9756]	24975	-0.760	-0.5553	Yes
92	CXCL8	C-X-C motif chemokine ligand 8 [Source:HGNC Symbol;Acc:HGNC:6025]	24983	-0.761	-0.5523	Yes
93	COL6A2	collagen type VI alpha 2 chain [Source:HGNC Symbol;Acc:HGNC:2212]	24984	-0.761	-0.5491	Yes
94	CAP2	cyclase associated actin cytoskeleton regulatory protein 2 [Source:HGNC Symbol;Acc:HGNC:20039]	25017	-0.766	-0.5469	Yes
95	CDH2	cadherin 2 [Source:HGNC	25052	-	-	Yes

		Symbol;Acc:HGNC:1759]		0.770	0.5448	
96	SGCG	sarcoglycan gamma [Source:HGNC Symbol;Acc:HGNC:10809]	25384	-0.813	-0.5522	Yes
97	MATN3	matrilin 3 [Source:HGNC Symbol;Acc:HGNC:6909]	25393	-0.814	-0.5490	Yes
98	NTM	neurotrimin [Source:HGNC Symbol;Acc:HGNC:17941]	25423	-0.819	-0.5465	Yes
99	SNTB1	syntrophin beta 1 [Source:HGNC Symbol;Acc:HGNC:11168]	25461	-0.824	-0.5443	Yes
100	BDNF	brain derived neurotrophic factor [Source:HGNC Symbol;Acc:HGNC:1033]	25500	-0.828	-0.5420	Yes
101	FZD8	frizzled class receptor 8 [Source:HGNC Symbol;Acc:HGNC:4046]	25576	-0.839	-0.5409	Yes
102	DAB2	DAB adaptor protein 2 [Source:HGNC Symbol;Acc:HGNC:2662]	25657	-0.849	-0.5400	Yes
103	COL3A1	collagen type III alpha 1 chain [Source:HGNC Symbol;Acc:HGNC:2201]	25779	-0.865	-0.5403	Yes
104	VCAM1	vascular cell adhesion molecule 1 [Source:HGNC Symbol;Acc:HGNC:12663]	25955	-0.892	-0.5423	Yes
105	TPM4	tropomyosin 4 [Source:HGNC Symbol;Acc:HGNC:12013]	26062	-0.908	-0.5419	Yes
106	ITGAV	integrin subunit alpha V [Source:HGNC Symbol;Acc:HGNC:6150]	26259	-0.934	-0.5444	Yes
107	TNFRSF11B	TNF receptor superfamily member 11b [Source:HGNC Symbol;Acc:HGNC:11909]	26564	-0.970	-0.5503	Yes
108	EFEMP2	EGF containing fibulin extracellular	26675	-	-	Yes

		matrix protein 2 [Source:HGNC Symbol;Acc:HGNC:3219]		0.981	0.5498	
109	TNFRSF12A	TNF receptor superfamily member 12A [Source:HGNC Symbol;Acc:HGNC:18152]	26855	-1.003	-0.5514	Yes
110	PLOD2	procollagen-lysine,2-oxoglutarate 5-dioxygenase 2 [Source:HGNC Symbol;Acc:HGNC:9082]	27088	-1.043	-0.5547	Yes
111	GADD45B	growth arrest and DNA damage inducible beta [Source:HGNC Symbol;Acc:HGNC:4096]	27305	-1.080	-0.5572	Yes
112	DCN	decorin [Source:HGNC Symbol;Acc:HGNC:2705]	27418	-1.099	-0.5562	Yes
113	MGP	matrix Gla protein [Source:HGNC Symbol;Acc:HGNC:7060]	27446	-1.103	-0.5525	Yes
114	RGS4	regulator of G protein signaling 4 [Source:HGNC Symbol;Acc:HGNC:10000]	27448	-1.104	-0.5478	Yes
115	GREM1	gremlin 1, DAN family BMP antagonist [Source:HGNC Symbol;Acc:HGNC:2001]	27515	-1.117	-0.5453	Yes
116	CD59	CD59 molecule (CD59 blood group) [Source:HGNC Symbol;Acc:HGNC:1689]	27519	-1.118	-0.5407	Yes
117	PMP22	peripheral myelin protein 22 [Source:HGNC Symbol;Acc:HGNC:9118]	27637	-1.142	-0.5397	Yes
118	FBLN1	fibulin 1 [Source:HGNC Symbol;Acc:HGNC:3600]	27746	-1.164	-0.5383	Yes
119	VEGFC	vascular endothelial growth factor C [Source:HGNC	27948	-1.205	-0.5398	Yes

		Symbol;Acc:HGNC:12682]				
120	PLAUR	plasminogen activator, urokinase receptor [Source:HGNC Symbol;Acc:HGNC:9053]	28183	-1.257	-0.5422	Yes
121	LOX	lysyl oxidase [Source:HGNC Symbol;Acc:HGNC:6664]	28299	-1.283	-0.5406	Yes
122	MXRA5	matrix remodeling associated 5 [Source:HGNC Symbol;Acc:HGNC:7539]	28358	-1.297	-0.5370	Yes
123	TGFBR3	transforming growth factor beta receptor 3 [Source:HGNC Symbol;Acc:HGNC:11774]	28367	-1.299	-0.5318	Yes
124	MAGEE1	MAGE family member E1 [Source:HGNC Symbol;Acc:HGNC:24934]	28428	-1.310	-0.5282	Yes
125	IL6	interleukin 6 [Source:HGNC Symbol;Acc:HGNC:6018]	28442	-1.312	-0.5231	Yes
126	LRP1	LDL receptor related protein 1 [Source:HGNC Symbol;Acc:HGNC:6692]	28496	-1.323	-0.5192	Yes
127	LRRC15	leucine rich repeat containing 15 [Source:HGNC Symbol;Acc:HGNC:20818]	28638	-1.359	-0.5181	Yes
128	FBN1	fibrillin 1 [Source:HGNC Symbol;Acc:HGNC:3603]	28679	-1.367	-0.5137	Yes
129	TNFAIP3	TNF alpha induced protein 3 [Source:HGNC Symbol;Acc:HGNC:11896]	28684	-1.368	-0.5080	Yes
130	TPM1	tropomyosin 1 [Source:HGNC Symbol;Acc:HGNC:12010]	28697	-1.370	-0.5026	Yes
131	MMP2	matrix metallopeptidase 2	28701	-	-	Yes

		[Source:HGNC Symbol;Acc:HGNC:7166]		1.372	0.4969	
132	ITGA5	integrin subunit alpha 5 [Source:HGNC Symbol;Acc:HGNC:6141]	28802	- 1.400	- 0.4943	Yes
133	SERPINE1	serpin family E member 1 [Source:HGNC Symbol;Acc:HGNC:8583]	28931	- 1.431	- 0.4925	Yes
134	ANPEP	alanyl aminopeptidase, membrane [Source:HGNC Symbol;Acc:HGNC:500]	28978	- 1.444	- 0.4879	Yes
135	COL5A2	collagen type V alpha 2 chain [Source:HGNC Symbol;Acc:HGNC:2210]	28984	- 1.445	- 0.4819	Yes
136	CRLF1	cytokine receptor like factor 1 [Source:HGNC Symbol;Acc:HGNC:2364]	28985	- 1.445	- 0.4758	Yes
137	THBS2	thrombospondin 2 [Source:HGNC Symbol;Acc:HGNC:11786]	29024	- 1.455	- 0.4709	Yes
138	COL1A2	collagen type I alpha 2 chain [Source:HGNC Symbol;Acc:HGNC:2198]	29030	- 1.455	- 0.4650	Yes
139	TIMP1	TIMP metallopeptidase inhibitor 1 [Source:HGNC Symbol;Acc:HGNC:11820]	29046	- 1.460	- 0.4593	Yes
140	NID2	nidogen 2 [Source:HGNC Symbol;Acc:HGNC:13389]	29078	- 1.467	- 0.4541	Yes
141	COL6A3	collagen type VI alpha 3 chain [Source:HGNC Symbol;Acc:HGNC:2213]	29093	- 1.472	- 0.4483	Yes
142	CDH11	cadherin 11 [Source:HGNC Symbol;Acc:HGNC:1750]	29242	- 1.527	- 0.4468	Yes
143	IGFBP4	insulin like growth factor binding protein	29265	-	-	Yes

		4 [Source:HGNC Symbol;Acc:HGNC:5473]		1.533	0.4410	
144	COL12A1	collagen type XII alpha 1 chain [Source:HGNC Symbol;Acc:HGNC:2188]	29274	-1.537	-0.4348	Yes
145	FBLN5	fibulin 5 [Source:HGNC Symbol;Acc:HGNC:3602]	29326	-1.554	-0.4299	Yes
146	BGN	biglycan [Source:HGNC Symbol;Acc:HGNC:1044]	29376	-1.572	-0.4249	Yes
147	CDH6	cadherin 6 [Source:HGNC Symbol;Acc:HGNC:1765]	29414	-1.584	-0.4194	Yes
148	PTX3	pentraxin 3 [Source:HGNC Symbol;Acc:HGNC:9692]	29455	-1.600	-0.4139	Yes
149	LOXL1	lysyl oxidase like 1 [Source:HGNC Symbol;Acc:HGNC:6665]	29478	-1.608	-0.4079	Yes
150	SERPINH1	serpin family H member 1 [Source:HGNC Symbol;Acc:HGNC:1546]	29486	-1.612	-0.4013	Yes
151	FMOD	fibromodulin [Source:HGNC Symbol;Acc:HGNC:3774]	29493	-1.613	-0.3947	Yes
152	COL4A1	collagen type IV alpha 1 chain [Source:HGNC Symbol;Acc:HGNC:2202]	29515	-1.621	-0.3885	Yes
153	SERPINE2	serpin family E member 2 [Source:HGNC Symbol;Acc:HGNC:8951]	29521	-1.626	-0.3818	Yes
154	FBLN2	fibulin 2 [Source:HGNC Symbol;Acc:HGNC:3601]	29545	-1.636	-0.3756	Yes
155	PCOLCE2	procollagen C-endopeptidase enhancer 2 [Source:HGNC Symbol;Acc:HGNC:8739]	29548	-1.637	-0.3688	Yes

156	FERMT2	fermitin family member 2 [Source:HGNC Symbol;Acc:HGNC:15767]	29559	-1.642	-0.3622	Yes
157	IL32	interleukin 32 [Source:HGNC Symbol;Acc:HGNC:16830]	29615	-1.668	-0.3569	Yes
158	THBS1	thrombospondin 1 [Source:HGNC Symbol;Acc:HGNC:11785]	29721	-1.722	-0.3531	Yes
159	FAP	fibroblast activation protein alpha [Source:HGNC Symbol;Acc:HGNC:3590]	29730	-1.725	-0.3461	Yes
160	INHBA	inhibin subunit beta A [Source:HGNC Symbol;Acc:HGNC:6066]	29764	-1.741	-0.3398	Yes
161	LAMC1	laminin subunit gamma 1 [Source:HGNC Symbol;Acc:HGNC:6492]	29777	-1.746	-0.3328	Yes
162	GJA1	gap junction protein alpha 1 [Source:HGNC Symbol;Acc:HGNC:4274]	29780	-1.748	-0.3255	Yes
163	SPARC	secreted protein acidic and cysteine rich [Source:HGNC Symbol;Acc:HGNC:11219]	29782	-1.749	-0.3182	Yes
164	COL4A2	collagen type IV alpha 2 chain [Source:HGNC Symbol;Acc:HGNC:2203]	29802	-1.761	-0.3113	Yes
165	GAS1	growth arrest specific 1 [Source:HGNC Symbol;Acc:HGNC:4165]	29812	-1.767	-0.3042	Yes
166	FSTL1	follistatin like 1 [Source:HGNC Symbol;Acc:HGNC:3972]	29821	-1.770	-0.2970	Yes
167	SLIT2	slit guidance ligand 2 [Source:HGNC Symbol;Acc:HGNC:11086]	29850	-1.786	-0.2903	Yes
168	RHOB	ras homolog family member B [Source:HGNC Symbol;Acc:HGNC:668]	29872	-1.795	-0.2834	Yes

169	JUN	Jun proto-oncogene, AP-1 transcription factor subunit [Source:HGNC Symbol;Acc:HGNC:6204]	29895	-1.809	-0.2765	Yes
170	SFRP4	secreted frizzled related protein 4 [Source:HGNC Symbol;Acc:HGNC:10778]	30016	-1.886	-0.2725	Yes
171	HTRA1	HtrA serine peptidase 1 [Source:HGNC Symbol;Acc:HGNC:9476]	30048	-1.910	-0.2654	Yes
172	TIMP3	TIMP metallopeptidase inhibitor 3 [Source:HGNC Symbol;Acc:HGNC:11822]	30061	-1.923	-0.2577	Yes
173	COL1A1	collagen type I alpha 1 chain [Source:HGNC Symbol;Acc:HGNC:2197]	30064	-1.924	-0.2497	Yes
174	TAGLN	transgelin [Source:HGNC Symbol;Acc:HGNC:11553]	30083	-1.934	-0.2421	Yes
175	FUCA1	alpha-L-fucosidase 1 [Source:HGNC Symbol;Acc:HGNC:4006]	30097	-1.943	-0.2343	Yes
176	FN1	fibronectin 1 [Source:HGNC Symbol;Acc:HGNC:3778]	30107	-1.953	-0.2263	Yes
177	AREG	amphiregulin [Source:HGNC Symbol;Acc:HGNC:651]	30111	-1.955	-0.2182	Yes
178	POSTN	periostin [Source:HGNC Symbol;Acc:HGNC:16953]	30178	-2.018	-0.2118	Yes
179	TPM2	tropomyosin 2 [Source:HGNC Symbol;Acc:HGNC:12011]	30189	-2.028	-0.2036	Yes
180	SCG2	secretogranin II [Source:HGNC Symbol;Acc:HGNC:10575]	30216	-2.053	-0.1958	Yes
181	ADAM12	ADAM metallopeptidase domain 12 [Source:HGNC Symbol;Acc:HGNC:190]	30254	-2.101	-0.1881	Yes

182	THY1	Thy-1 cell surface antigen [Source:HGNC Symbol;Acc:HGNC:11801]	30272	-2.122	-0.1797	Yes
183	MYLK	myosin light chain kinase [Source:HGNC Symbol;Acc:HGNC:7590]	30281	-2.132	-0.1710	Yes
184	CXCL12	C-X-C motif chemokine ligand 12 [Source:HGNC Symbol;Acc:HGNC:10672]	30285	-2.139	-0.1620	Yes
185	COL11A1	collagen type XI alpha 1 chain [Source:HGNC Symbol;Acc:HGNC:2186]	30318	-2.186	-0.1538	Yes
186	CALD1	caldesmon 1 [Source:HGNC Symbol;Acc:HGNC:1441]	30345	-2.219	-0.1453	Yes
187	ACTA2	actin alpha 2, smooth muscle [Source:HGNC Symbol;Acc:HGNC:130]	30351	-2.227	-0.1361	Yes
188	ABI3BP	ABI family member 3 binding protein [Source:HGNC Symbol;Acc:HGNC:17265]	30361	-2.241	-0.1269	Yes
189	LUM	lumican [Source:HGNC Symbol;Acc:HGNC:6724]	30417	-2.352	-0.1188	Yes
190	NT5E	5'-nucleotidase ecto [Source:HGNC Symbol;Acc:HGNC:8021]	30420	-2.363	-0.1088	Yes
191	SFRP1	secreted frizzled related protein 1 [Source:HGNC Symbol;Acc:HGNC:10776]	30428	-2.378	-0.0990	Yes
192	CCN2	cellular communication network factor 2 [Source:HGNC Symbol;Acc:HGNC:2500]	30445	-2.419	-0.0893	Yes
193	MFAP5	microfibril associated protein 5 [Source:HGNC Symbol;Acc:HGNC:29673]	30455	-2.439	-0.0793	Yes

194	APLP1	amyloid beta precursor like protein 1 [Source:HGNC Symbol;Acc:HGNC:597]	30459	-2.465	-0.0690	Yes
195	EDIL3	EGF like repeats and discoidin domains 3 [Source:HGNC Symbol;Acc:HGNC:3173]	30461	-2.467	-0.0586	Yes
196	VCAN	versican [Source:HGNC Symbol;Acc:HGNC:2464]	30513	-2.605	-0.0493	Yes
197	CCN1	cellular communication network factor 1 [Source:HGNC Symbol;Acc:HGNC:2654]	30534	-2.721	-0.0385	Yes
198	NNMT	nicotinamide N-methyltransferase [Source:HGNC Symbol;Acc:HGNC:7861]	30553	-2.832	-0.0271	Yes
199	MYL9	myosin light chain 9 [Source:HGNC Symbol;Acc:HGNC:15754]	30608	-3.449	-0.0143	Yes
200	COMP	cartilage oligomeric matrix protein [Source:HGNC Symbol;Acc:HGNC:2227]	30610	-3.496	0.0005	Yes
